# Association of COVID-19 mortality with serum selenium, zinc and copper: Six observational studies across Europe

**DOI:** 10.3389/fimmu.2022.1022673

**Published:** 2022-11-28

**Authors:** Kamil Demircan, Thilo Samson Chillon, Tommy Bracken, Ilaria Bulgarelli, Irene Campi, Gijs Du Laing, Samira Fafi-Kremer, Laura Fugazzola, Alejandro Abner Garcia, Raban Heller, David J. Hughes, Louis Ide, Georg Jochen Klingenberg, Pawel Komarnicki, Zbigniew Krasinski, Alain Lescure, Patrick Mallon, Arash Moghaddam, Luca Persani, Mirko Petrovic, Marek Ruchala, Morgane Solis, Linos Vandekerckhove, Lutz Schomburg

**Affiliations:** ^1^ Institute for Experimental Endocrinology, Charité-Universitätsmedizin Berlin, and Berlin Institute of Health, Berlin, Germany; ^2^ School of Medicine, University College Dublin, Dublin, Ireland; ^3^ Laboratorio Analisi Cliniche, Centro di Ricerche e Tecnologie Biomediche, IRCCS Istituto Auxologico Italiano, Milano, Italy; ^4^ Division of Endocrine and Metabolic Diseases, Istituto Auxologico Italiano, Istituto Di Ricovero e Cura a Carattere Scientifico (IRCCS), Milan, Italy; ^5^ Laboratory of Analytical Chemistry and Applied Ecochemistry, Faculty of Bioscience Engineering, Ghent University, Gent, Belgium; ^6^ CHU de Strasbourg, Laboratoire de Virologie, Strasbourg University, INSERM, IRM UMR-S 1109, Strasbourg, France; ^7^ Department of Pathophysiology and Transplantation, University of Milan, Milan, Italy; ^8^ Centre for Experimental Pathogen Host Research, School of Medicine, University College Dublin, Dublin, Ireland; ^9^ Clinic of Traumatology and Orthopaedics, Bundeswehr Hospital Berlin, Berlin, Germany; ^10^ Department of General Practice and Health Services Research, Heidelberg University Hospital, Heidelberg, Germany; ^11^ School of Biomolecular and Biomedical Science, UCD Conway Institute, University College Dublin, Dublin, Ireland; ^12^ Laboratory Medicine, AZ Jan Palfijn AV, Gent, Belgium; ^13^ Department of Endocrinology, Metabolism, and Internal Diseases, Poznan University of Medical Sciences, Poznan, Poland; ^14^ Department of Vascular and Endovascular Surgery, Angiology and Phlebology, Poznan University of Medical Sciences, Poznan, Poland; ^15^ Architecture et Réactivité de l’ARN, CNRS, Université de Strasbourg, Strasbourg, France; ^16^ Orthopedic and Trauma Surgery, Aschaffenburg, Germany; ^17^ Department of Medical Biotechnology and Translational Medicine, University of Milan, Milan, Italy; ^18^ Department of Internal Medicine and Paediatrics, Ghent University, Gent, Belgium

**Keywords:** trace elements, SARS-CoV-2, mortality, biomarker, nutrition

## Abstract

**Introduction:**

Certain trace elements are essential for life and affect immune system function, and their intake varies by region and population. Alterations in serum Se, Zn and Cu have been associated with COVID-19 mortality risk. We tested the hypothesis that a disease-specific decline occurs and correlates with mortality risk in different countries in Europe.

**Methods:**

Serum samples from 551 COVID-19 patients (including 87 non-survivors) who had participated in observational studies in Europe (Belgium, France, Germany, Ireland, Italy, and Poland) were analyzed for trace elements by total reflection X-ray fluorescence. A subset (n=2069) of the European EPIC study served as reference. Analyses were performed blinded to clinical data in one analytical laboratory.

**Results:**

Median levels of Se and Zn were lower than in EPIC, except for Zn in Italy. Non-survivors consistently had lower Se and Zn concentrations than survivors and displayed an elevated Cu/Zn ratio. Restricted cubic spline regression models revealed an inverse nonlinear association between Se or Zn and death, and a positive association between Cu/Zn ratio and death. With respect to patient age and sex, Se showed the highest predictive value for death (AUC=0.816), compared with Zn (0.782) or Cu (0.769).

**Discussion:**

The data support the potential relevance of a decrease in serum Se and Zn for survival in COVID-19 across Europe. The observational study design cannot account for residual confounding and reverse causation, but supports the need for intervention trials in COVID-19 patients with severe Se and Zn deficiency to test the potential benefit of correcting their deficits for survival and convalescence.

## 1 Introduction

The trace elements copper (Cu), selenium (Se) and zinc (Zn) are essential dietary micronutrients and exert essential roles in the organism (1–3), including proper immune function (2–6). All three elements are central constituents of regulatory or structural proteins such as selenoproteins, zinc proteins or cuproproteins, affecting a wide range of signaling and biochemical pathways, and cell activities (7, 8).

The dietary intakes of several micronutrients varies across the world, with particularly strong differences known for Cu, Se, Fe, I, and Zn (9–11). Moreover, supply might change with future climatic alterations, causing further risks of deficiency (12). Especially during pregnancy, a supply below the Recommended Dietary Allowance (RDA) is of particular concern as it may negatively affect both mother health and child development (13–15). Additional requirements and strong alterations are observed in disease, particularly in critical illness (16, 17). The fast decline in circulating Se and Zn is proposed to constitute a physiological meaningful reaction that may turn problematic for recovery during longer stays in the intensive care unit and during convalescence (18, 19). Accordingly, the issue of substitution or supplementation in pregnancy and disease as an adjuvant measure against low status and developing deficiency has been intensively discussed (20–23). A general supplementation in the preventive setting without diagnosed deficiency, for reducing for example cardiovascular disease or cancer risk, is hitherto not recommended (24), but a personalized correction of severe deficits appears meaningful in subjects with insufficient supply or in severe disease (25–27).

The coronavirus disease 2019 (COVID-2019) pandemic is challenging the intensive care units around the world with many critically diseased patients and a high death toll. Severe disease is associated with strongly altered serum trace element concentrations, notably low concentrations of Se (28–30) and Zn (29–32), and slightly or strongly elevated serum Cu (32, 33). The observed changes are in line with prior reports on the acute phase response in infection or sepsis, where serum Se is established as a negative (34–36), and serum Cu as a positive acute phase reactant (37, 38). Response of Zn seems more complex as it involves strong and dynamic redistribution between the tissues (39). On a population-wide scale, cure rate and death toll have been reported to correlate with soil Se and population biomarkers of Se status in China, where Se availability varies strongly around the country (40, 41).

Considering that different European populations have diverse dietary patterns and habitual intake levels of essential micronutrients, in particular of Se and Zn, we decided to compare trace element status in six European observational studies in relation to COVID-19 mortality. We hypothesized to observe characteristic alterations in serum trace elements in COVID-19 patients across the countries in comparison to healthy European subjects, suitable to derive a population-independent model and relevant thresholds with predictive value for survival.

## 2 Materials and methods

### 2.1 Overview of cohorts, samples and laboratory analyses

All subjects enrollment into the study and analyses of their biosamples were conducted in accordance with the Declaration of Helsinki. Ethical approval for the study was obtained from the appropriate ethical committees in each European country. All patients or their relatives provided written informed consent prior to the analyses. An overview of the different cohorts is given in **Supplementary Table 1**. Blood was drawn by venipuncture, serum or plasma samples were prepared by standard operating procedures, aliquoted and stored at -80°C until they were sent on dry ice to the analytical lab at Charité-Universitätsmedizin Berlin in Germany. Measurements of all samples included in this manuscript were conducted by scientists and technicians blinded to the clinical information, in the same laboratory using the same technique (TXRF). A total of 860 samples with corresponding trace element measurements were available for the 551 patients, and were included in the main analyses. For Belgium, 143 samples with a median (IQR) of 2 (2,2) samples per patients were available, for France 124 samples with median (IQR) of 3 (2,4), for Germany 169 samples with median (IQR) of 4 (2,6), for Poland 153 samples with median (IQR) of 1 (1,1) per patient were collected, while one sample only was collected for each patient during hospital admission in Ireland and Italy.

#### 2.1.1 Belgium, CO-vim

The Belgian CO-vim study and the analysis of the trace elements have been described before (42). Serum samples of patients with COVID-19 were collected at Ghent University Hospital (UZ Gent) and AZ Jan Palfijn Hospital (JPH Ghent) in Belgium as described (42). Ethical counselling was provided by the local Ethics Committee of JPH Ghent and UZ Gent, and approval was granted (BC-07492).

#### 2.1.2 France, COVID-HUS

The COVID-HUS study is a monocentric observational longitudinal cohort study enrolling COVID-19 patients admitted at the Strasbourg University Hospitals, as described before (43). The study was approved by the institutional review boards of the Strasbourg University Hospitals (Clinical Trials.gov identifier: NCT04405726).

#### 2.1.3 Germany, TASC-3B

The study and the analysis of the trace elements have been described before (28). It was a cross-sectional study including surviving and non-surviving patients with COVID-19 hospitalized at Klinikum Aschaffenburg-Alzenau in Germany. Ethical counselling had been obtained from the authorities in Bavaria (Ethik-Kommission der Bayerischen Landesärztekammer, EA No. #20033), and the study was registered at the German Clinical Trial Register (Deutsches Register Klinischer Studien, ID: DRKS00022294).

#### 2.1.4 Ireland, AIIDC

The All Ireland Infectious Diseases Cohort Study is a prospective, multicentric study, enrolling COVID-19 patients, as described before (44, 45). The study was approved by local institutional review boards.

#### 2.1.5 Italy, COV-endo

The Italian COV-endo study is a prospective cohort study involving COVID-19 patients admitted to the COVID-19 Units of San Luca Hospital, Istituto Auxologico Italiano in Milan, Italy. Elaboration on the study details has been described before (46, 47). The study was approved by the ethics committee of the institution (COV-endo study, n 05C021).

#### 2.1.6 Poland, ENDO-COVID-PL

Patients were hospitalized at regional hospital in Słupca, Wielkopolskie, Poland. Analysis is a part of the ongoing project “Analysis of trace elements, Vitamin D, ghrelin, TSH, thyroid hormones, hepcidin and irisin within population of patients hospitalized due to COVID-19 in regional hospital in Słupca, Poland”. Bioethical Committee at Poznan University of Medical Sciences was consulted for the purpose of this study. Due to analysis of archived and anonymized samples as well as retrospective character of the research no Bioethical Committee agreement was deemed necessary according to bioethical regulations in Poland. Statement signed by the head of Bioethical committee was acquired.

#### 2.1.7 European Prospective Investigation into Cancer and Nutrition

A cross sectional overview of serum trace element concentrations was obtained by an analysis of serum samples from subjects participating in the European Prospective Investigation into Cancer and Nutrition (EPIC) study (48, 49). A total of 2069 healthy adult subjects from the different participating countries had been analyzed by the same technology as part of a prospective study on colorectal cancer (50). All study participants provided written informed consent. Local review boards of each participating center as well as review boards of the International Agency for Research on Cancer (IARC) provided ethical approval. Reference ranges for serum Cu, Se and Zn concentrations were deduced according to the range between 2.5^th^ and 97.5^th^ centiles of all samples analyzed.

### 2.2 Trace element quantification

Serum samples were diluted 1:2 with a standard solution containing 1000 µg/L Gallium. An aliquot of the dilution (8 µL) was applied to quartz glass slides (Bruker nano, Berlin, Germany), and dried. Analysis of trace elements was conducted by total reflection X-ray fluorescence (TXRF) spectroscopy by an ultra-trace element analysis system (S4 T-STAR, Bruker nano, Berlin, Germany) as described (51, 52). A serum standard was measured with each measurement, and intra- and inter-assay coefficients of variation were below 8% at all times.

### 2.3 Statistical analyses

#### 2.3.1 Descriptive statistics

Continuous data was described using median (IQR), and categorical data was described as number (percent). Density plots were applied to evaluate and display distribution of continuous data, separately for each cohort where applicable.

#### 2.3.2 Evaluation of serum biomarker interdependence

Two statistical methods were applied to assess the interrelationship between the biomarkers. First, a common dimension reduction method comprising the principal component analysis (53) (PCA) was applied in order to depict an unsupervised overview of the trace element patterns. Principal components accounting for the highest explanation of variation were deduced, considering scree plots as well as eigenvalues above one. Biplots were used to display the patterns. Secondly, correlation of the trace elements were assessed separately using Spearman’s rank correlation test. Correlation coefficients (Spearman’s R) derived were presented separately for each combination of trace element for every cohort including the reference cohort. Scatter plots were used to visualize overall correlations.

#### 2.3.3 Trace element status in relation to reference

Reference for the trace element status was derived from a healthy subset of the EPIC-study, comprising 2069 individuals from different European countries. 2.5^th^ and 97.5^th^ centiles for each trace element were calculated, which served as reference range. Presence of trace element deficiencies in COVID-19 patients was calculated according to this range and combined deficiencies were presented as Venn diagrams.

#### 2.3.4 Trace element status in relation to death

Concentrations of each trace element were compared between samples of deceased and surviving patients for each country separately using Wilcoxon-rank sum test to detect crude differences between the two groups. In a pooled analysis of all patients in each study, logistic regression with three restricted cubic splines (RCS) adapted at three predefined knots (10^th^, 50^th^, and 90^th^ percentile) was conducted to assess the relationship between trace element concentrations and death. First samples of the patients after hospital admission were considered in the analyses. Shape of association was evaluated by comparing the RCS models with their respective linear regression models *via* likelihood ratio X^2^ test. Computed *p nonlinearity* below 0.05 was considered as a nonlinear relationship. All models were adjusted for age, sex and country. Receiver operating characteristic (ROC) analyses were conducted, and areas under the curves (AUC) were computed to serve as discrimination indices for comparison of the predictive value for different trace elements.

All statistical analyses were performed using the R language (version 4.1.2) on the R Studio Integrated Development Environment. *dplyr* and *tidyr* packages were used to manipulate data structures and data. *ggplot2, ggpubr, ggbiplot, ggvenn, maps, ggridges, ggstatsplot, ggbiplot, gtsummary, flextable and rms* packages were used to generate plots and compute statistical analyses.

## 3 Results

**Figure 1:**
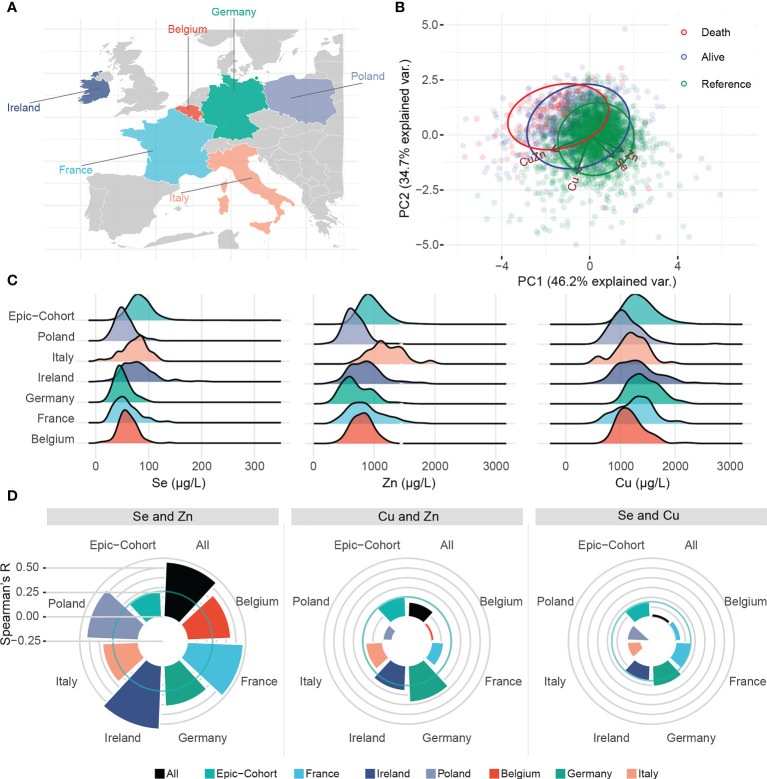
Trace element patterns and correlations. **(A)** Map displaying the countries contributing to this study. **(B)** Biplot of principal component analysis displaying patterns of trace elements, and Cu/Zn ratio in relation to clusters of alive, deceased and healthy subjects. **(C)** Ridgeline plot displaying the distribution of each trace element in each country as well as the healthy EPIC reference. **(D)** Spearman’s R for correlation between trace elements in each country. Solid circular green lines represent the correlations in the reference group, i.e. a representative sample of the EPIC-Study.

A total of 551 hospitalized COVID-19 positive patients were enrolled in six studies across six European countries. Patient characteristics are presented in **Table 1**. Median age was similar across the countries, with a median above 60 years. Among 551 patients, a total of 87 deaths were recorded. Ten deaths occurred in Belgium, 14 deaths in France, six deaths in Germany, 14 deaths in Ireland, 10 deaths in Italy and 33 deaths in Poland (**Figure 1A**).

**Table 1:** Age and sex of patients enrolled stratified by country.

	Belgium	France	Germany	Ireland	Italy	Poland
Characteristic	n = 74	n = 44	n = 35	n = 227	n = 44	n = 127
Age	62 (52, 71)	70 (61, 76)	77 (64, 82)	61 (47, 75)	66 (56, 73)	72 (63, 77)
Sex						
Female	23 (31%)	13 (30%)	20 (57%)	118 (52%)	10 (23%)	62 (49%)
Male	51 (69%)	31 (70%)	15 (43%)	109 (48%)	34 (77%)	65 (51%)

Median (IQR); n (%).

The Cu, Se, and Zn status of COVID-19 positive patients were compared with 2069 healthy adults participating in the cross-sectional EPIC-study (European Prospective Investigation into Cancer and Nutrition). These analyses were conducted in the same laboratory as the other measurements reported here, using the same method. For the reference range the 2.5^th^ and 97.5^th^ centiles of the EPIC-reference were calculated which corresponded to 45.9 µg/L and 132.4 µg/L for Se, 640.9 µg/L and 1469.5 µg/L for Zn, and 894.2 µg/L and 2098.9 µg/L for Cu, respectively.

A principal component analysis (PCA) was conducted to assess potential trace element patterns in patients deceased from COVID-19, surviving patients and the reference group (**Figure 1B** **)**. Se and Zn followed a similar pattern, while Cu exhibited a distinct pattern. In **Figure 1C**, we assessed the distribution of trace elements in each country and in the reference cohort. When assessing correlations, in line with the PCA analysis, Se and Zn correlated positively in the reference cohort (Spearman’s R = 0.261) (**Figure 1D**). The correlation coefficient of Se and Zn in COVID-19 patients was more than two-fold higher than in the reference group, consistently in each country (overall Spearman’s R = 0.564). The correlation of Se and Cu or Cu and Zn were positive overall, but not consistent across all countries.

**Figure 2:**
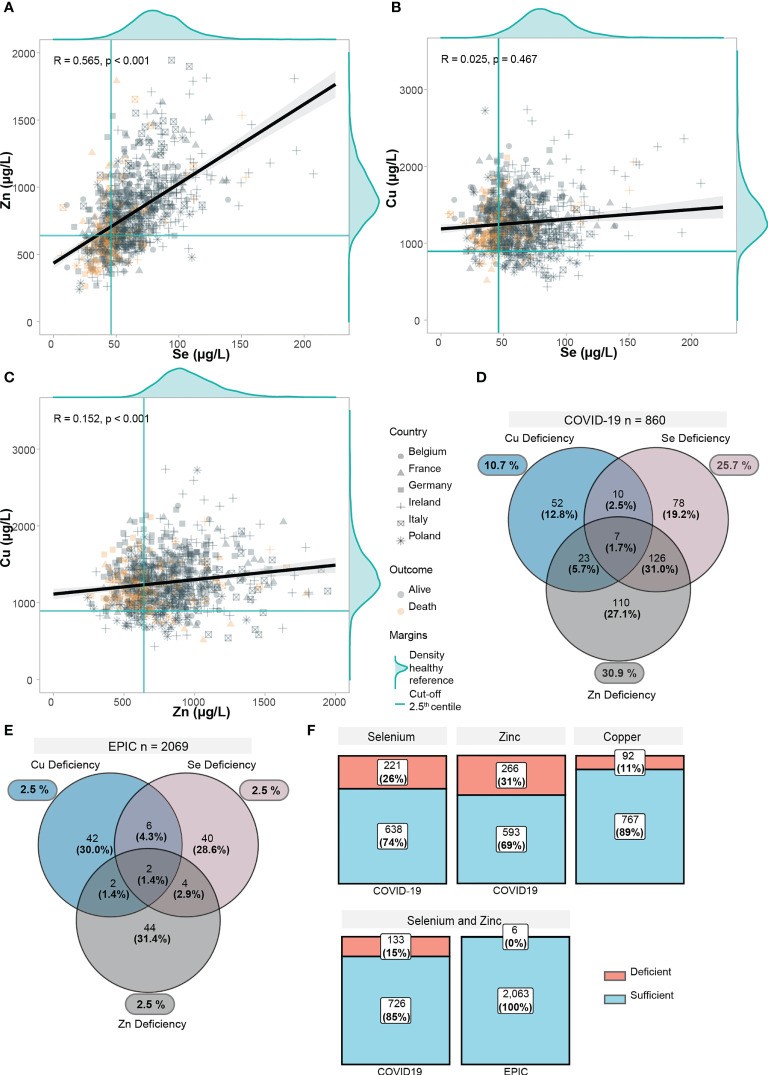
Trace element status in relation to each other and EPIC-reference cohort. **(A–C)** Scatter plots displaying the correlation between trace elements in COVID-19 positive patients. Yellow points display deceased patients, grey points display alive patients. Point shapes differ according to each country. For each trace element, the distribution of the healthy EPIC-cohort (n=2069) was plotted as marginal density plots. Green lines correspond to the 2.5^th^ lowest centile in the EPIC-cohort. Black line is computed using linear regression, gray shadows correspond to 95% confidence intervals. Correlation coefficient (R) was computed with the Spearman’s R. **(D)** Deficiencies in trace elements (below 2.5^th^ centile in EPIC) are shown. Venn diagram shows overlapping/combined deficiencies. **(E)** Deficiencies in trace elements are shown in the EPIC cohort. **(F)** Proportions of deficiency in each trace element as well as a combined Se and Zn deficiency are displayed.

2.5^th^ centiles correspond to green solid lines in **Figures 2A–C**, and overall distribution of the EPIC-reference is displayed as marginal density of the scatter plots. Overall correlations of the trace elements were displayed in scatter plots in **Figures 2A–C**. According to the 2.5^th^ centiles of the reference cohort, 10.7% of the samples were Cu deficient, 25.7% were Se deficient, and 30.9% displayed Zn deficiency (**Figure 2D**). Combined deficiencies were most prominent for Se and Zn, as 32.7% of the deficient samples also presented an underlying combined deficiency. In comparison, combined deficiencies were rare (0.3% for a combined Se and Zn deficiency) in the EPIC cohort (**Figures 2E, F**).

**Figure 3:**
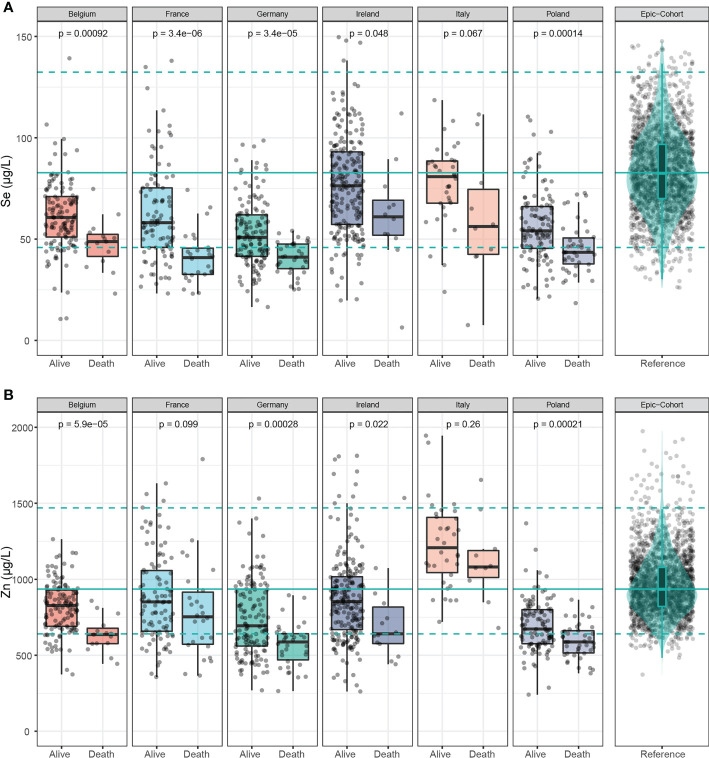
Selenium and Zn status in samples of deceased and alive patients. **(A)** Serum Se concentrations were lower in samples of non-survivors than in survivors across all countries. **(B)** Serum Zn concentrations were also relatively low in the non-survivors across all countries. The green line denotes the median value as determined in EPIC, and broken lines indicate the 2.5th and 97.5th centiles. Results from Wilcoxon-Rank-sum testing are indicated as p-values.

In **Figure 3A**, serum Se concentrations were compared between samples of surviving and deceased patients in each country. Consistent throughout all six countries, Se concentrations were lower in the samples of deceased patients, and the findings were statistically significant, except for Italy, where a trend was observed, however without reaching statistical significance. Similarly, Zn concentrations were significantly lower in the samples of the non-survivors, except for France and Italy (**Figure 3B**).

**Figure 4:**
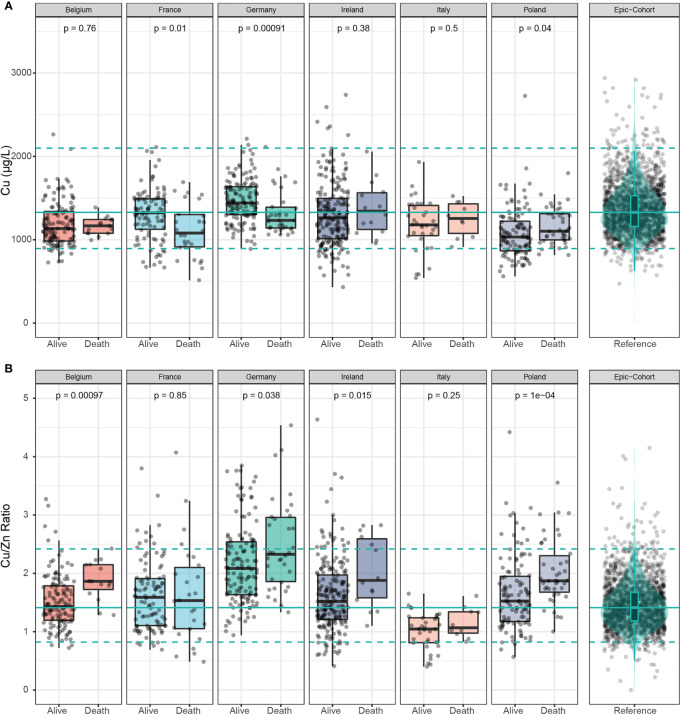
Cu and Cu/Zn status in samples of deceased and alive patients. **(A)** Serum Cu concentrations showed inconsistent differences between samples of deceased and alive patients across all countries. **(B)** The serum Cu/Zn ratios was relatively higher in non-survivors than in survivors in all the countries with France as the exception. The green line denotes the median value as determined in EPIC, and broken lines indicate the 2.5^th^ and 97.5^th^ centiles. Results from Wilcoxon-Rank-sum testing are indicated as p-values.

Serum Cu was significantly lower in samples of non-survivors in France and Germany, but displayed significantly higher concentrations in samples of deceased patients in Poland (**Figure 4A**). Cu/Zn ratio was consistently elevated in deceased patients across four of the countries, except for Italy and France (**Figure 4B**).

An analysis of the country-specific values highlights relatively consistent differences between the surviving and non-surviving patients for serum Se and Zn, but not for Cu. Serum Se concentrations were 19.1-30.4% lower in samples of non-survivors than in survivors, and serum Zn concentrations were 10.5-24.7% lower in samples of the patients who died as compared to those who survived (**Table 2**).

**Table 2:** Relative differences in serum Cu, Se and Zn with respect to survival.

Country	Cu (alive/death)*	Se (alive/death)*	Zn (alive/death)*
Belgium	2.9	-19.6	-22.9
France	-18.4	-29.6	-11.5
Germany	-14.4	-19.1	-15.3
Ireland	5.9	-17.2	-24.7
Italy	6.7	-30.4	-10.5
Poland	6.8	-19.6	-12.6

* (in %), calculated as -((median(alive)-median(death))/median(alive) * 100).

**Figure 5:**
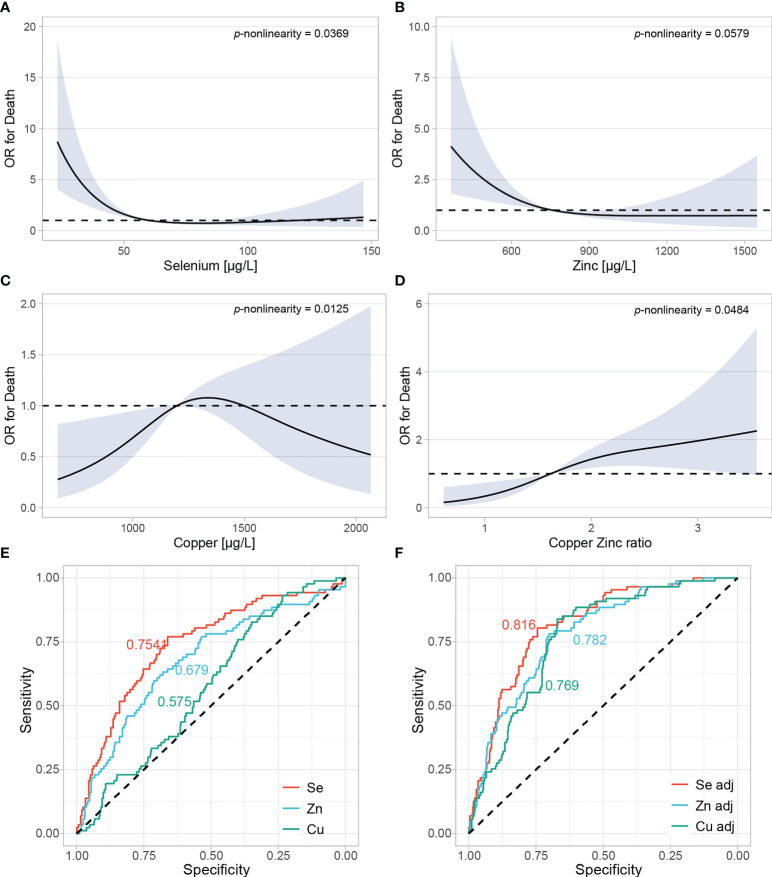
Adjusted restricted cubic spline regression in a pool of all patients from all countries. **(A–D)** Restricted cubic spline regression with three knots at 10^th^, 50^th^ and 90^th^ centiles were conducted to assess the relationship between trace elements and odds ratio (OR) of death. All analyses were adjusted for age, sex, and country of the samples. *P* for *nonlinearity* was calculated by comparing nested linear regression models to the restricted cubic splines by likelihood ratio X^2^ test. **(E)** Se, Zn and Cu were evaluated as individual predictors in relation to death using the restricted cubic spline model. Areas under the curves (AUC) are plotted for each predictor. **(F)** Models were further adjusted for age and sex.

A pooled analysis based on adjusted restricted cubic spline regression was conducted to assess the relationship between trace element concentrations and death from COVID-19 (**Figure 5**). The models were further adjusted for age, sex, and country of the patients included (n=549, two missing due to missingness in age). Se and Zn displayed a significant, inverse, non-linear relationship with death from COVID-19 (**Figures 5A, B**). Low Cu was associated with a favorable outcome (**Figure 5C**), while the ratio of Cu to Zn displayed a positive association with death (**Figure 5D**). In order to assess and compare the potential predictive value of each marker, we conducted receiver operating characteristics analyses. As individual predictors, Se had the highest AUC of 0.754, followed by Zn (0.679), and Cu (0.575) (**Figure 5E**). Combined models considering age and sex displayed an AUC of 0.816 for Se, 0.782 for Zn, and 0.769 for Cu (**Figure 5F**). 164 female and 54 male COVID-19 patients with a median (IQR) age of 44 (33,53) were additionally enrolled as outpatients into the AIIDC study in Ireland. When associating trace element concentrations with hospitalization, considering the outpatients as the control group, the results were similar to those with death as an endpoint (**Supplementary Figure 1**). After adjusting for age and sex, Se and Zn were inversely, nonlinearly associated with hospitalization (**Supplementary Figures 1A, B**). Cu was not associated with hospitalization, but the ratio of Cu to Zn was linearly and positively associated with hospitalization (**Supplementary Figures 1C, D**). Zn had the highest predictive value (AUC of 0.658), followed by Se (0.625) (**Supplementary Figure 1E**). In this analysis, Cu did not perform better than chance (AUC of 0.499). After including patients’ age and sex, the model including Zn had an AUC of 0.790, model with Se had an AUC of 0.789, and the model with Cu displayed an AUC of 0.777 (**Supplementary Figure 1F**).

## 4 Discussion

In this study, we assessed serum Cu, Se and Zn concentrations in COVID-19 patients from separate observational studies conducted in six European countries. A general deficiency in serum Se and Zn in comparison to a reference range from 2069 healthy European adults was observed in the patients, in particular in the non-survivors. Serum Cu concentrations showed no consistent difference, neither in comparison to the reference nor in relation to survival, whereas the Cu/Zn ratio was particularly high in non-survivors, presumably largely attributable to low Zn status.

A parallel regulation of Se and Zn has been observed in the control subjects, much in agreement with another independent sample (n=3834) of the EPIC cohort analyzed recently by a different technology (54). Notably, correlation analyses of the two different sub-cohorts including almost 6000 participants of EPIC yielded identical coefficients for serum Se and Zn, of 0.26 (54) and 0.261 (this study). The almost perfect accordance supports the robustness of this interaction, as it was found in two large subsamples of EPIC in different laboratories using different analytical techniques (inductively coupled plasma tandem mass spectrometry (ICP-MS/MS) versus TXRF). The correlation for serum Se and Zn showed a substantially higher coefficient in the patient samples as compared to EPIC, highlighting common COVID-19-related pathways apparently regulating these two immune-relevant trace elements in the same direction. However, this notion is deduced from single samples only, taken mainly at hospital admission. Our previous time-resolved analyses highlighted low serum Se as part of the acute phase response, causing further selenoprotein P decline with time especially in the non-survivors (28). In comparison, the decline of Zn appears to be a fast reaction and rather a re-distribution (39), as the concentrations tended to normalize with time both in survivors and non-survivors (31).

A regular metabolism and sufficient supply of Se and Zn are of central importance for a full activity of the immune system. The trace element Zn is needed for the function of hundreds of different proteins in our organism, influences the activity and response of immune cells, directly serves signal transduction, and apparently also alters the composition of the microbiota (55–57). Moreover, Zn status is reduced in the elderly, which is associated with a declining activity of the immune system (58). Although this association implies a protective role of Zn against COVID-19 mortality, the results of observational studies are rather ambiguous and support the quest for targeted intervention studies to answer this hypothesis (59, 60). The analysis of an open-label supplementation study with Zn and Se in patients with severe COVID-19 induced acute respiratory distress syndrome (ARDS) supports the hypothesis of positive health effects on the immune system, but results from sufficiently large and randomized controlled intervention studies are lacking (61). The understanding of the molecular effects of Se deficiency and supplemental Se intake on the immune system is more solid, as the Se status directly affects the expression of selenoproteins relevant to the immune system (62–64). The link extents to multiple levels, including quality control of newly synthesized proteins in the ER to prevent secretion of immunogenic misfolded proteins (65, 66), a direct involvement of selenoproteins in intracellular lymphocyte signaling (67–69), and an effect of selenoproteins, particularly GPX4, on immune cell survival and protection against hyperactivation–induced ferroptosis (70, 71). Given that severe Se deficiency is an established risk factor for autoimmune disease (72–74) and appears to influence mortality risk in critical illness (20, 75, 76), the consistent associations observed in this study support discussion of active supplementation of COVID-19 patients with severe deficiency to achieve reference concentrations (19). The extent to which a combined supplementation of Se and Zn in severe COVID-19 has a positive effect on disease progression, immune system function, and survival remains to be investigated. However, based on what is known from supplementation studies, it is unlikely that in patients with diagnosed deficiency, correcting circulating concentrations towards reaching reference ranges will cause adverse side effects in a given patient.

To the best of our knowledge, this is the first multi-cohort study assessing several trace elements across different countries by standardized methodology in one analytical laboratory. The results from two of the six studies have been reported before (Belgium and Germany), where additional biomarkers were determined, verifying the differences also by longitudinal alterations and in different comorbidities (28, 31, 33, 42). Due to reasons of limited sample volumes from some cohorts, differences in the pre-analytical sample preparations, and in view that our prior studies reported consistent results for additional biomarkers of trace element status, this study focused on a single parameter using one robust technique, i.e., total concentrations determined by TXRF analysis.

When compared to other studies, our observations are in line with the findings from several single centers regarding the relatively low concentrations of serum Se and Zn in COVID-19 and in particular in relation to survival (30, 77–81). Beside individual data level analyses, geographical soil trace element content has also been assessed in relation to COVID-19 mortality, yielding congruent results with our study (40, 41). In the USA however, where soil Se content is higher than in European and many Asian and African countries, an association of the case fatality rate of COVID-19 with geography was observed for Zn only, but not for Se (82). Considering that the majority of the US population is Se replete, this finding is indeed reasonable and argues for a potential causal relationship.

Beside serum concentrations, cumulative population level or geographical data, the dietary intake and genetically predicted trace element status has also been assessed in relation to COVID-19 mortality. In contrast to the analytical findings with serum samples, no significant associations were observed for Se, Cu or Zn in genetic analyses based on Mendelian Randomization (MR) studies (83, 84).

Using the EPIC-cohort as reference, serum Se and Zn was lower in the COVID-19 patients analyzed. The relative difference to EPIC was comparable between the countries, despite the relatively high Zn status in Italy, which likely results from locally preferred dietary patterns and food availability (85). In general, vegetarians and vegans have poor supply of Se and Zn, in agreement with the low concentration in soil and accordingly in local plant products (86–88). A diet rich in fish, seafood and meat provides higher amounts of trace elements, as they tend to accumulate in the sea and are usually added to the feed of chicken, pigs or cattle in the farming process (89, 90). Hence, some of the country-specific differences observed may be attributable to the fraction of vegetarians and the frequency of meat and fish-containing meals, however no dietary intake data have been determined in the current study.

Our study has several strengths and limitations. We collected patient samples in six countries of Europe, establishing a relatively large cohort, and conducting a parallel quantitative analysis blinded to clinical data during the measurements and by the same technology. Hereby, the risk of personal bias and technological errors are minimized. Moreover, we were able to relate the findings to a similar analysis of a large sub cohort of healthy European subjects from the EPIC study (50). The congruent results from the PCA for serum Se and Zn support the notion on a representative database and reliable technology (50, 54). The parallel decline of Se and Zn, notably in non-survivors, supports the clinical relevance of the changes for survival prediction. The findings support the notion on the need for conducting micronutrient supplementation to avoid severely declined status and health-compromising deficiencies, as both elements are known for their essential role in the immune system and for convalescence (91, 92).

However, due to the observational study design, we cannot account for reverse causality, i.e., disease severity may have suppressed serum trace element status, with the changes observed potentially not affecting the course of the disease. Another notable limitation concerns the heterogeneity of the studies. Even though all six studies investigated COVID-19 mortality as a main outcome, time point of sampling, clinical care, potential adjuvant treatments, age and sex range of the patients included in the analyses differed between the study sites. This considerable limitation among others precluded on the one hand a reliable adjustment of the analyses for additional clinical parameters, e.g., comorbidities, medication and other potential confounders, so that we cannot account for residual confounding. Yet, the high consistency of the results on the other hand despite these considerable sources of heterogeneity highlights and underlines the robustness of the major results, providing a high generalizability of our findings.

This study supports the notion on a mortality-relevant decline of serum Se and Zn concentrations in COVID-19, irrespective of area of residency in Europe and country specific dietary patterns. As Europe is generally characterized by relatively low Se supply, the findings should not be extrapolated to areas of higher habitual Se intake, from where similar analyses have not yet been reported. As our observational study design cannot address reverse causality, supplementation recommendations are limited to the consented importance of avoiding severe deficiencies, but are not yet based on clinical experience as appropriately designed intervention trials with Se and/or Zn are missing in COVID-19. In case the strong deficits in nonsurvivors contributed to death risk, a personalized substitution for raising the status into the range of survivors would constitute a straightforward, inexpensive and promising adjuvant treatment option, which merits serious consideration as a testable hypothesis.

## Data availability statement

The datasets presented in this article are not readily available because availability is based on varying local governmental restrictions for each country. R code for statistical analyses conducted to generate the main findings in this study will be made available upon request. Requests to access the datasets should be directed to lutz.schomburg@charite.de.

## Ethics statement

The studies involving human participants were reviewed and approved by Local Ethics Committee of JPH Ghent and UZ Gent, Institutional review boards of the Strasbourg University Hospitals, Bavaria Ethik-Kommission der Bayerischen Landesärztekammer, Local Irish institutional review boards, ethics committee of Istituto Auxologico Italiano in Milan, Italy, Bioethical Committee at Poznan University of Medical Sciences. The patients/participants provided their written informed consent to participate in this study.

## Author contributions

KD: conceptualization, methodology, software, formal analysis, investigation, data curation, writing - original draft, and visualization; TC: conceptualization, methodology, software, formal analysis, investigation, data curation, writing - original draft, and visualization; TB: conceptualization, resources, and writing - review and editing; IB: conceptualization, resources, and writing - review and editing; IC: conceptualization, resources, and writing - review and editing; GDL: conceptualization, resources, and writing - review and editing; SF-K: conceptualization, resources, and writing - review and editing; LF: conceptualization, resources, and writing – review and editing; AG: conceptualization, resources, and writing - review and editing; RH: writing – review and editing and investigation; DH: conceptualization, resources, and writing - review and editing; LI: conceptualization, resources, and writing - review and editing; GK: writing – review and editing, investigation, and methodology; PK: conceptualization, resources, and writing - review and editing; ZK: conceptualization, resources, and writing - review and editing; AL: conceptualization, resources, and writing - review and editing; PM: conceptualization, resources, and writing - review and editing; AM: conceptualization, resources, and writing – review and editing; LP: conceptualization, resources, and writing – review and editing; MP: conceptualization, resources, and writing - review and editing; MR: conceptualization, resources, and writing - review and editing; MS: conceptualization, resources, and writing – review and editing; LV: conceptualization, resources, and writing - review and editing; LS: conceptualization, validation, resources, writing – original draft, supervision, project administration, and funding acquisition. All authors contributed to the article and approved the submitted version.

## Funding

This work was supported by the Deutsche Forschungsgemeinschaft (DFG), Research Unit FOR-2558 “TraceAge” (Scho 849/6–2) and CRC/TR 296 “Local control of TH action” (LocoTact, P17), and partially by the Italian Ministry of Health (COVENDO study).

## Acknowledgments

The authors would like to thank Vartitér Seher, Gabriele Boehm and Anja Fischbach for excellent technical support. Jozefien De Clercq, Jana Minne and Laura Van der Meulen for their contribution to data collection and processing, the All-Ireland Infectious Diseases (AIID) Cohort Study Group for support, and Rafał Spachacz and Bożena Barczyk from regional hospital in Słupca for excellent cooperation in acquiring and processing of the samples.

## Conflict of interest

LS holds shares of selenOmed GmbH, a company involved in selenium status assessment.

The remaining authors declare that the research was conducted in the absence of any commercial or financial relationships that could be construed as a potential conflict of interest.

## Publisher’s note

All claims expressed in this article are solely those of the authors and do not necessarily represent those of their affiliated organizations, or those of the publisher, the editors and the reviewers. Any product that may be evaluated in this article, or claim that may be made by its manufacturer, is not guaranteed or endorsed by the publisher.
