# Supplementary material for: Association of COVID-19 mortality with serum selenium, zinc and copper: Six observational studies across Europe
Source: Front Immunol. 2022 Nov 28;13:1022673. doi: 10.3389/fimmu.2022.1022673 (PMC9742896; doi:10.3389/fimmu.2022.1022673)
Supplement: Supplementary file 1 [file DataSheet_1.pdf]

Supplementary Material

Supplementary Table 1. Relevant background information by cohort

		**TASC-3B**
**Cohort**	Full Name	Traceelement ATORG Study COVID-19 in Berlin, Bavaria and Baden-Württemberg
**Germany**	Description	Characterization of COVID-19 infections using immune system parameters and trace element status.
	References/ID	ID: DRKS00022294
	Contact	Prof. Lutz Schomburg
	Funding Information	Budgetary funds of Klinikum Aschaffenburg-Alzenaugemeinnützige GmbH, no external funding
		**Co-Vim**
**Cohort**	Full Name	Cohort 1: UZ Gent (Co-Vim) Virological and Immunological Monitoring in Patients (suspected of/confirmed with) COVID-19 – Cohort 2: AZ Jan Palfijn: Course and survival of COVID-19 patients with comorbidities in relation to the trace element status at hospital admission
**Belgium**	Description	Cross-sectional observational study conducted at two hospitals in Ghent, Belgium, to investigate whether Se and/or Zn deficiency upon hospital admission correlates to disease severity and mortality risk in COVID-19 patients with or without co-morbidities. Trace element concentrations along with additional biomarkers were determined in serum or plasma and associated to disease severity and outcome.
	References/ID	BC-07492
	Contact	Prof. Gijs Du Laing, Mirko Petrovic
	Funding Information	Budgetary funds of Ghent University, UZ Ghent and AZ Jan Palfijn, no external funding
		**COVID-HUS**
**Cohort**	Full Name	COVID- Hôpitaux Universitaires de Strasbourg (HUS)
**France**	Description	The COVID-HUS study is a monocentric observational longitudinal cohort study enrolling COVID-19 patients admitted at the Strasbourg University Hospitals.
	References/ID	NCT 04405726
	Contact	Prof. Alain Lescure, Samira Fafi-Kremer
	Funding Information	This study was supported by the Strasbourg University Hospital (COVID-HUS study-HUS N 7760)
		**COV-endo study**
**Cohort**	Full Name	COVENDO
**Italy**	Description	Characterization of the endocrine dysfunctions associated with covid-19 infection in hospitalized patients
	References/ID	n 05C021_2020
	Contact	Prof. Luca Persani
	Funding Information	The work was partially supported by the Ricerca Corrente Funds of Istituto Auxologico Italiano (Acronym: COV-endo study, n 05C021_2020)
		**ENDO-COVID-PL**
**Cohort**	Full Name	COVID-19: Analysis of Vitamin D, ghrelin and other neuropeptides over the course of SARS-CoV-2 infection.
**Poland**	Description	Analysis of trace elements, Vitamin D, ghrelin, TSH, thyroid hormones, hepcidin and irisin with population hospitalized due to COVID-19 in regional hospital in Słupca, Poland.
	References/ID	Regional biooethical committee agreement – 19.01.2022

	Contact	Paweł Komarnicki, Marek Ruchala, Zbigniew Krasiński
	Funding Information	Department of Endocrinology, Metabolism and Internal Disease, Poznań University of Medical Sciences

		AIID
**Cohort**	Full Name	All Ireland Infectious Diseases Study
**Ireland**	Description	The All Ireland Infectious Diseases Study is a prospective multicentric study, enrolling COVID-19 patients.
	References/ID	The AIID Cohort is approved by local institutional review boards and all participants provide written, informed consent.
	Contact	David J. Hughes, Patrick Mallon
	Funding Information	Science Foundation Ireland under the Science Foundation Ireland, Enterprise Ireland, IDA Ireland COVID-19 Rapid Response Funding Call (Grant number: COVID-RRC 20/COV/0103 and COVID-RRC 20/COV/0305).

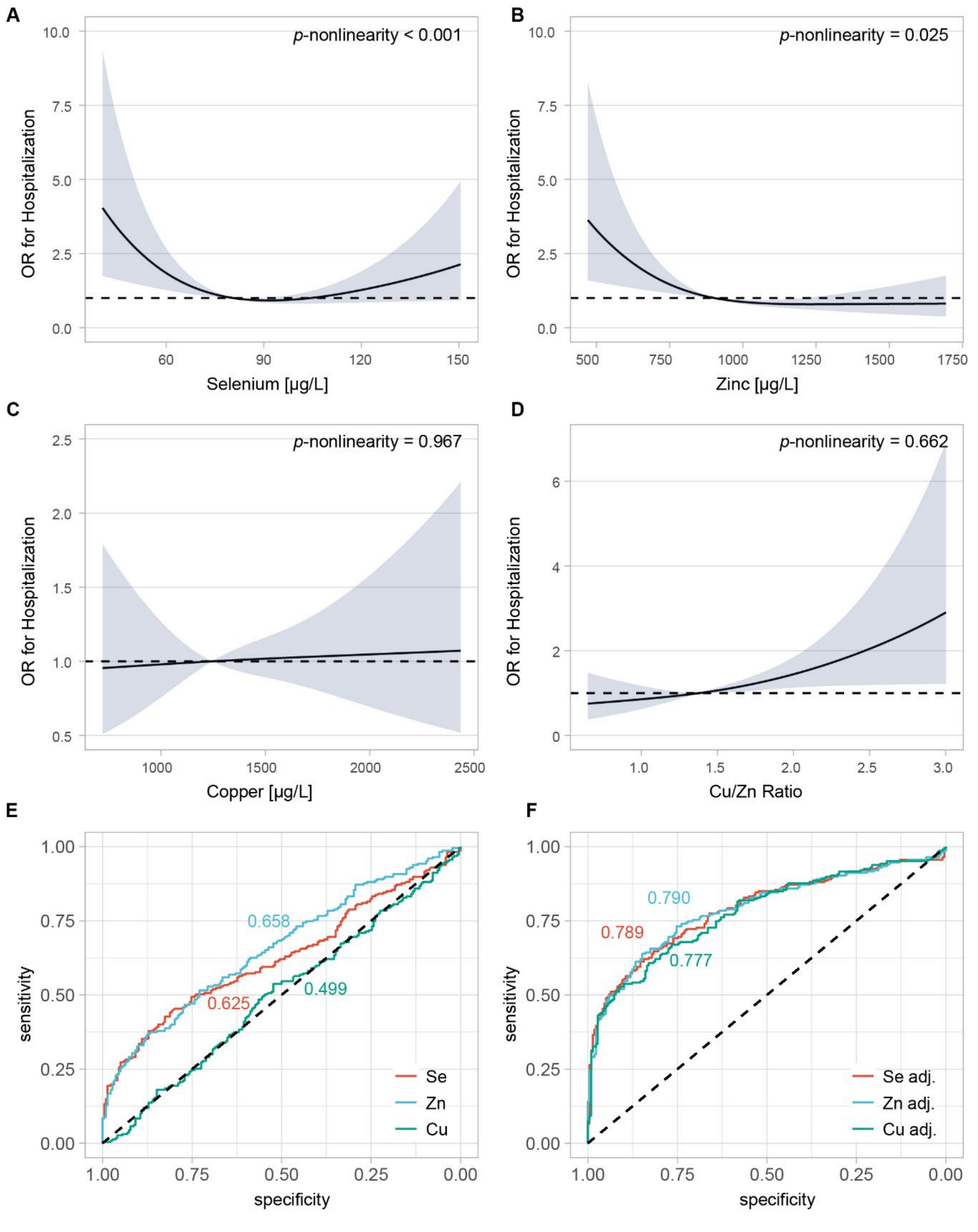

Supplementary Figure 1. Adjusted restricted cubic spline regression for the odds ratio of hospitalization in the Ireland study. A,**B**,**C**,**D** Restricted cubic spline regression with 3 knots at 10th, 50th and 90th centiles were conducted to assess the relationship between trace elements and odds ratio (OR) of hospitalization. All analyses were adjusted for age and sex of the patients. *P* for *nonlinearity* was calculated by comparing nested linear regression models to the restricted cubic splines by likelihood ratio X^2 test. **E** Se, Zn and Cu were evaluated as individual predictors in relation to hospitalization using the restricted cubic spline model. Areas under the curves (AUC) are plotted for each predictor. **F** Models were further adjusted for age and sex.
